# Low-Latency Wireless Network Extension for Industrial Internet of Things

**DOI:** 10.3390/s24072113

**Published:** 2024-03-26

**Authors:** Michael Fletcher, Eric Paulz, Devin Ridge, Alan J. Michaels

**Affiliations:** Virginia Tech National Security Institute, Blacksburg, VA 24060, USA

**Keywords:** industrial IoT, low latency, RA-CDMA, spread spectrum, TSN

## Abstract

The timely delivery of critical messages in real-time environments is an increasing requirement for industrial Internet of Things (IIoT) networks. Similar to wired time-sensitive networking (TSN) techniques, which bifurcate traffic flows based on priority, the proposed wireless method aims to ensure that critical traffic arrives rapidly across multiple hops to enable numerous IIoT use cases. IIoT architectures are migrating toward wirelessly connected edges, creating a desire to extend TSN-like functionality to a wireless format. Existing protocols possess inherent challenges to achieving this prioritized low-latency communication, ranging from rigidly scheduled time division transmissions, scalability/jitter of carrier-sense multiple access (CSMA) protocols, and encryption-induced latency. This paper presents a hardware-validated low-latency technique built upon receiver-assigned code division multiple access (RA-CDMA) techniques to implement a secure wireless TSN-like extension suitable for the IIoT. Results from our hardware prototype, constructed on the IntelFPGA Arria 10 platform, show that (sub-)millisecond single-hop latencies can be achieved for each of the available message types, ranging from 12 bits up to 224 bits of payload. By achieving one-way transmission of under 1 ms, a reliable wireless TSN extension with comparable timelines to 802.1Q and/or 5G is achievable and proven in concept through our hardware prototype.

## 1. Introduction

The adoption of wired time-sensitive networking (TSN) techniques has taken hold over the last decade to support an explosion of factory automation, automated vehicles, motion control, and broadly emerging industrial Internet of Things (IIoT) applications. In particular, the 802.1 family of IEEE standards defines TSN-based networking requirements, protocols (802.1Q [1]), and timing synchronization (802.1AS, [2]) in support of low-latency/low-jitter communications within wired Ethernet networks. In general, these TSN-based networks employ gate control lists (GCLs) and other prioritization schemes to ensure the deterministic delivery of a small portion of the overall network traffic, supporting a rapid expansion of time-critical wired applications. Recent research has produced hardware testbeds implementing these TSN standards and achieved repeatable end-to-end latency performance of the order of 500-1000 µs for randomly generated audio/video bridging (AVB) packets [3] to as little as single-hop latencies of 6.4 µs with 802.1Qbv [1] and the IEEE-1588 Precision Timing Protocol (PTP) for in-vehicle communication systems [4]. These testbeds and related experiments help to solidify the expectations for using wired Ethernet backbones to transmit priority data on sub-millisecond time scales.

The 802.1Q TSN standards extend and codify prior (mostly proprietary) real-time Ethernet fieldbus standards such as the Process Field Net Isochronous Real-Time (PROFINET IRT, [5]), Ethernet for Control Automation Technology (EtherCAT, [6]), and the Serial Real-time Communication System (SERCOS III, [7]). These standards generally support response latencies of less than 1 ms and jitter of less than 100 µs while achieving aggregate fieldbus data rates of the order of 100 Mbps. A common characteristic among all of these TSNs is that they are physically tethered by wires [8], consistent with 94% of industrial networks over the past few years [9].

As newer IIoT applications are conceived, there is a strong desire to eliminate the wiring to edge nodes while retaining the latency and jitter performance assurances (or similar) as the 802.1Q TSN. Taken as a wireless sensor network (WSN), most of these edge nodes (ENs) connect through one (or a small number) of access points (APs) that aggregate data to/from the decision agent on a core network. These EN sensors are often tasked with collecting small repetitive data content (temperature, pressure, voltages, logical states) and reporting anomalous conditions/periodic heartbeats, which is achievable with data rates of 1–10 kbps per link. Moreover, command and control (C&C) information passed from the AP to the EN population is also low, with both sides of the link being limited by available bandwidth, associated channel capacity, and the underlying contention characteristic of the chosen multiple access scheme.

Many such sensors may be grouped into device classes that are bounded by 10 kbps steady-state data and burst modes of up to 100 kbps [10,11]. Various prior extensions of timing-sensitive data transfer have been proposed for WSNs, with early work on the time division multiple access (TDMA)-based 802.15.4 Zigbee standard [12] such as [13] proposing intelligent adaptations of the 802.15.4 guaranteed timeslot (GTS) mechanism after using carrier-sense multiple access with collision avoidance (CSMA/CA) for network formation; this 802.15.4 modification remains latency-challenged due to its inherent TDMA foundation and has achieved best-case latencies of 35 ms. More recent work evaluated topology-constrained wireless personal area networks using the frequency division multiple access (FDMA)-based 802.15.1 Bluetooth standard [14,15] and was able to achieve a latency range of 8–15 ms. Other ongoing work explored improvements of statistical priority-based multiple access (SPMA) [16] and achieved 500 µs for a single-hop latency; yet, the associated hardware and processing are considerably more complex than those supported by most IIoT edge nodes.

In recent years, updates to the IEEE 802.11 standard [17] have been proposed to support TSN functionality in IoT scenarios [18,19,20,21,22,23,24]. Specifically, 802.11be [25] and 802.11ax [26] seek to address worst-case jitter and latency while still achieving the high data rates supported by Wi-Fi. A variety of research has shown these modifications to support the sub-1 ms one-way latencies required to be compatible with TSN; however, 802.11-based technology is too power hungry for deployment to low-power edge nodes as is needed in many IIoT applications. Additional research within 5G supports the definition and prototyping of ultra-reliable low-latency communications (URLLCs), with future protocols having potential latencies as low as 1 ms for extremely short data payloads [19,27,28,29,30]. While a newer technology than Wi-Fi, the results are certainly promising and research has shown compatibility between 5G and existing IEEE TSN standards by incorporating a 5G/TSN bridge in the hybrid wired/wireless network. Similar to Wi-Fi, 5G technology is challenging to incorporate into battery-powered edge nodes, with many such designs requiring a cabled power source even though 5G successfully eliminates much of the bulky cabled network in IIoT.

A catalog of recent research works is provided in Table 1 to support the discussion. The focus of this literature search was any potential wireless TSN extension research within the past 3–5 years. Note that, in addition to 802.11 and 5G research, prior work has also focused on some lower-power, lower-data-rate technologies such as Bluetooth Low Energy (BLE) [31], wireless synchronous and hybrid architecture for real-time performance (w-SHARP) [32], and Digital Enhanced Cordless Telecommunications–Ultra-Low Energy (DECT-ULE) [33]. While the latency and jitter are relatively low in BLE networks, they are comparatively limited in range and face scalability problems [34,35]. The FDMA- and TDMA-based channel access methods in the other technologies also face scalability issues when compared to the wireless TSN technology proposed in this paper [36].

This paper demonstrates a custom arbitrary-phase spread spectrum physical layer (PHY) and MAC layer designed to implement a low-latency TSN-like wireless extension, i.e., a hybrid network, where highly scalable unscheduled data transmissions are transmitted within an RA-CDMA framework and connected into a wired core network. These results build upon prior work [36,37] comparing scalability (communications performance, latency, and jitter) between the time-slotted 802.15.4 and a custom RA-CDMA high-order phase shift keying (PSK) signaling (HOPS) waveform [38] when used in IIoT applications, yet have recently been implemented in hardware of an Arria 10 SoC device and subsequently translated to an Artix 7 100T device. The overall performance of these unscheduled waveforms yields one-way latencies for low-duty-cycle/short-burst messages of the order of 200–500 µs, with per-node user data rates of ≈70 kbps. Moreover, the scalability of the wireless TSN extension supports in excess of 100 nodes within each distinct subnet, leveraging a potential 4x network scalability advantage over alternative technologies [36] in addition to improved interference mitigation and security, as are common for spreading spectrum systems.

The main advantages of the proposed technology are summarized as follows:

- RA-CDMA networks have a scalability advantage over other multiple access protocols, driven largely by the inherent CDMA properties of spread spectrum signals.
- No scheduling is required in RA-CDMA since the transmission may begin as soon as data are available. Collisions only occur if times of arrival from different nodes are within three to five spreading chips (300–500 ns) of each other.
- The inherent security of dynamic (time-evolving) spreading codes means that the additional latency of cryptographic processing is not required [39,40].
- Sub-millisecond latency and very low jitter support TSN-like functions in the IIoT.
- Lower power requirements than 802.11 and 5G support battery-powered wireless TSN operations.

Details of the wireless TSN extension design are provided in Section 2, focusing on asynchronous transmission, data rates, and latency drivers. An analysis of associated timelines, design parameters, and implementation details is then provided in Section 3. Quantitative performance metrics for the hardware testbed are then provided in Section 4, followed by a brief presentation of limitations in Section 5 and conclusions with descriptions of future work in Section 6.

## 2. Framework for TSN in RA-CDMA Networks

**Figure 1:**
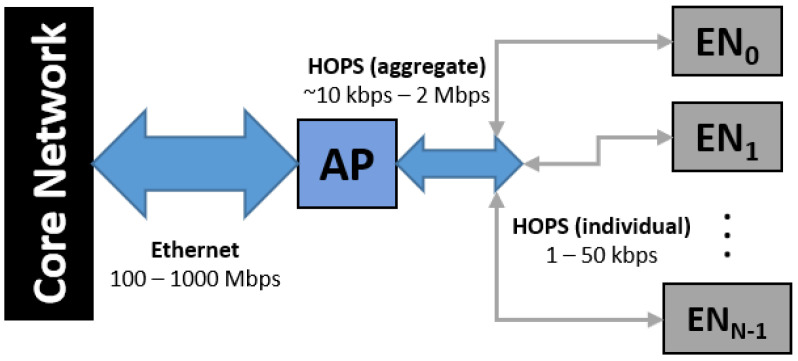
High-level network topology of HOPS integrated into a larger network.

In constructing a hybrid TSN-like extension, the largest design constraint to recognize is the relative disparity in data rates between the wired and wireless network extension. In effect, most wired Ethernet networks operate at line rates of at least 100–1000 Mbps, giving them a two to three order of magnitude advantage over wireless networks in delivering data, which may have individual links at 10 kbps and aggregate to a few Mbps, yet represent up to 90% of actual links as the edge layer in a hierarchical industrial setting. These figures assume an efficient reduction of data to only those required and would not support massive parallelized streams of real-time video (most of which is never consumed by humans). A conceptual snapshot of this data aggregation is shown in Figure 1.

Viewing the APs as data aggregators or wireless routers, the uplink traffic $d_{UL_{k}}$ from wireless sensor *k* to the core network is assumed to have minimal latency and/or contention beyond the wireless channel. Likewise, the capacity of the wireless channel and multiple access addressing scheme will dominate what may be transmitted as a downlink $d_{DL_{k}}$ from the core network to the nodes. The latency considerations for node *k* primarily have to do with any processing required from sensor value collection to framing as a wireless message ($\tau_{MAC}$), the delay between requesting and initiating transmission ($\tau_{Sched}$), the duration of the data frame ($\tau_{UL_{K}}$/$\tau_{DL_{K}}$), and any required AP processing, such as forward error correction (FEC) or adjudicating network priorities ($\tau_{RX}$). Of particular benefit for the RA-CDMA networks over TDMA-based timeslotted networks (802.15.4 and 5G) is that there is no scheduling of any kind, so messages are sent as soon as available; as a result, $\tau_{Sched}=0$.

**Figure 2:**
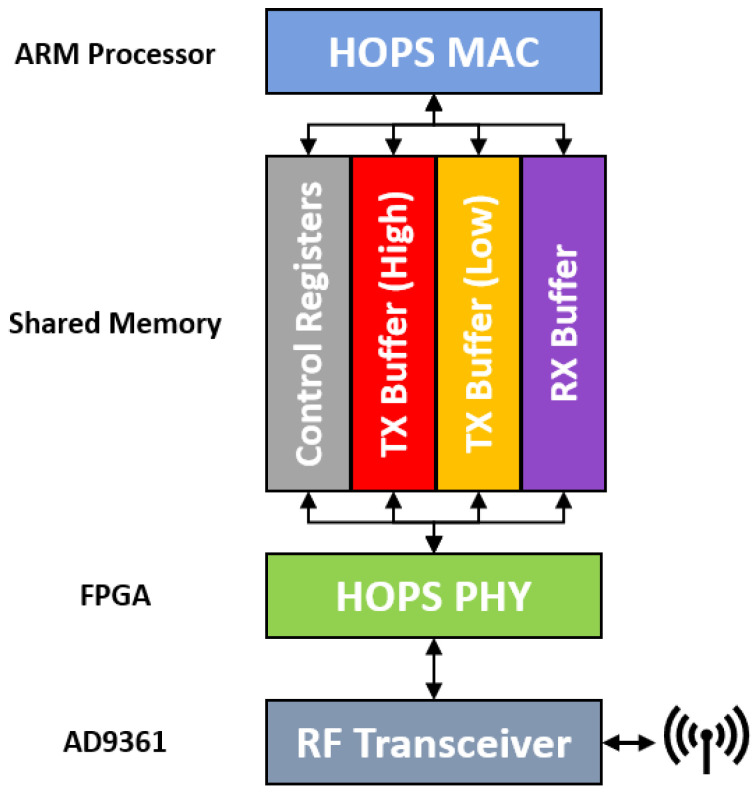
High-level HOPS System diagram as implemented on Intel Arria 10 platform.

The most common mechanism in wired TSN frameworks to ensure minimum latency for priority traffic is to implement two or more priority queues that are managed for port congestion [41]. For our hardware prototype, we implemented a two-level priority scheme within each wireless node. This priority scheme relies on coordination between our custom MAC software, which runs on the ARM processor as a C executable, and the HOPS PHY, which runs on the field-programmable gate array (FPGA) fabric. These two components communicate through a custom memory-mapped register interface where both the MAC and PHY can access control registers as well as circular buffers designated for transmit and receive data. The transmit buffer is separated into two partitions: one for high-priority outgoing traffic and the other for low priority. In order for both components to track the contents of each buffer, a set of pointer indices is maintained and updated by its respective component when a read or write operation is performed. For example, if the MAC writes a frame to be transmitted to the high-priority transmit buffer, it will subsequently increment variable TXMAC-HIGH. In the next cycle, the FPGA will compare TXMAC-HIGH to TXPHY-HIGH and see that the MAC layer has sent a new frame for transmission. The FPGA logic will always check the high-priority transmit buffer before anything else to ensure the timely delivery of critical messages. The same pointer logic applies to the low-priority transmit buffer and the receive buffer. Figure 2 provides a high-level system diagram showing the components described above.

**Figure 3:**
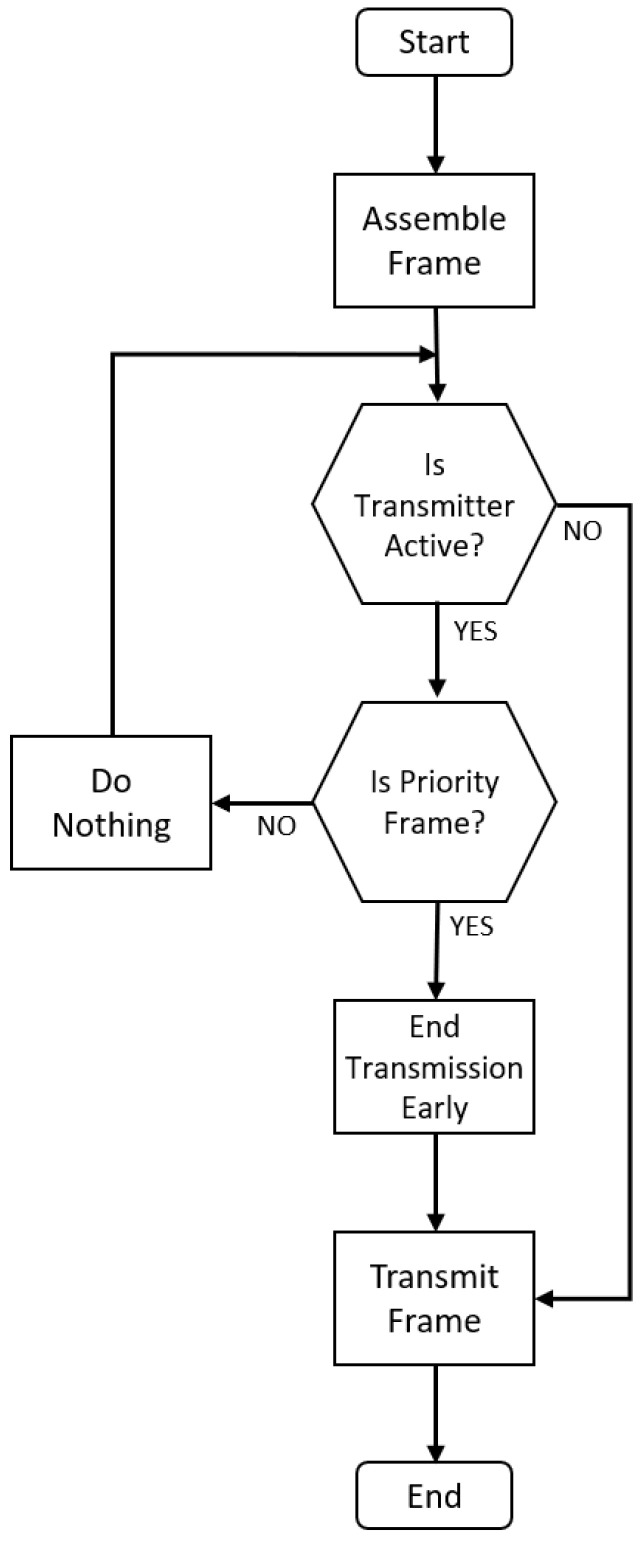
Flow chart representing the processing of priority frames.

Each individual message within the HOPS device is assigned a priority value within the MAC header. These MAC-layer message definitions include custom allocations to support short, medium, and long variations in both C&C and user data traffic. The multi-tier priority hierarchy eases development by ensuring that messages marked as high-priority get sent to the appropriate buffer automatically. Since the FPGA always checks the high-priority transmit buffer first, critical messages are always transmitted immediately (beginning at the next clock cycle) regardless of what operations are occurring in the background. This alleviates some processing duties at the receiver, which simply processes frames in the order that they are received while ensuring priority tags are maintained. A visual representation of this priority frame processing is shown in Figure 3. The primary difference between the presented message processing flow and others presented in the literature is the unique ability to terminate an outgoing low-priority message in favor of sending a high-priority one. Due to the nature of RA-CDMA, the terminated message may be instantly re-scheduled to be sent at the conclusion of the high-priority transmission. As such, the exact structure of the queuing is not the subject of this figure, since that is highly application-dependent, and may feature more than two queues.

## 3. System Design Parameters and Implementation Details

Armed with the HOPS design specification as well as a deep understanding of the platform capabilities, we can reasonably predict how the system will perform under the testing of time-sensitive functionality. This section provides key parameters and other information about the system and implementation specifics, which enable the highly scalable wireless network being presented.

The custom RA-CDMA MAC employs a discrete set of message sizes that are driven by a configurable polar-code-based FEC encoder/decoder core. Polar codes are systematic codes that have increased in popularity lately because they can perform well enough for short block codes at the expense of a generally acceptable reduction in performance (2.8 dB gap to the normal approximation bound) given their superior algorithmic complexity (one to six orders of magnitude fewer) [42]. These codes typically employ block sizes of integer powers of two (that is, $N=2^{b}$, b≥3). All messages employ an eight-symbol binary PSK (BPSK)-encoded preamble followed by message sizes selected from {15,31,63,127,255} bits to be quadrature PSK (QPSK)-encoded for a total of {8,16,32,64,128} symbols. The hardware prototypes employ a chip rate of 10 MHz and a spread ratio of 175, corresponding to a symbol duration of 17.5 µs. Moreover, the AP contains multiple demodulator cores to support simultaneous reception from different ENs.

The various message types, FEC parameters, and transmission latencies ($\tau_{UL_{k}}/\tau_{DL_{k}}$) are all shown in Table 2. The corresponding calculated durations for min- and max-size messages are 280 µs and 2.38 ms, respectively. The reception processing ($\tau_{RX}$) is driven by the FEC decoder latency; processing delays range from 14.4 µs to 494 µs using the 20 MHz clock (note that a 100 or 200 MHz clock may easily be used here for a 5–10*x* improvement). In our hardware example, we only implemented a single FEC decoder instance, so there was a potential backlog from the multi-demodulator receiver during high network activity. These baseline delays can be further exacerbated by the inherent complexity of the custom MAC layer and system as a whole, which is why it is important to carefully consider which messages should be marked as critical and which should not. Priority assignment can change based on the application at hand but, fundamentally, the designer should consider which messages could lead to unsafe operation of the network if not received and processed immediately.

To use an example from the existing HOPS message set, a node operating as a secondary user in a shared spectrum may detect a primary user in the RF environment and broadcast a message to the rest of the HOPS network to shut down all communications and enter emissions control (EMCON) mode. This type of message will govern the practical co-existence with the primary user, ultimately setting the tempo for non-contentious use of the spectrum. Likewise, certain C&C traffic is considered high-priority, such as commands to adjust the transmit power level or center frequency, as well as any messages pertinent to transmission security (TRANSEC) operation (e.g., toggling hopping states or updating keys).

**Figure 4:**
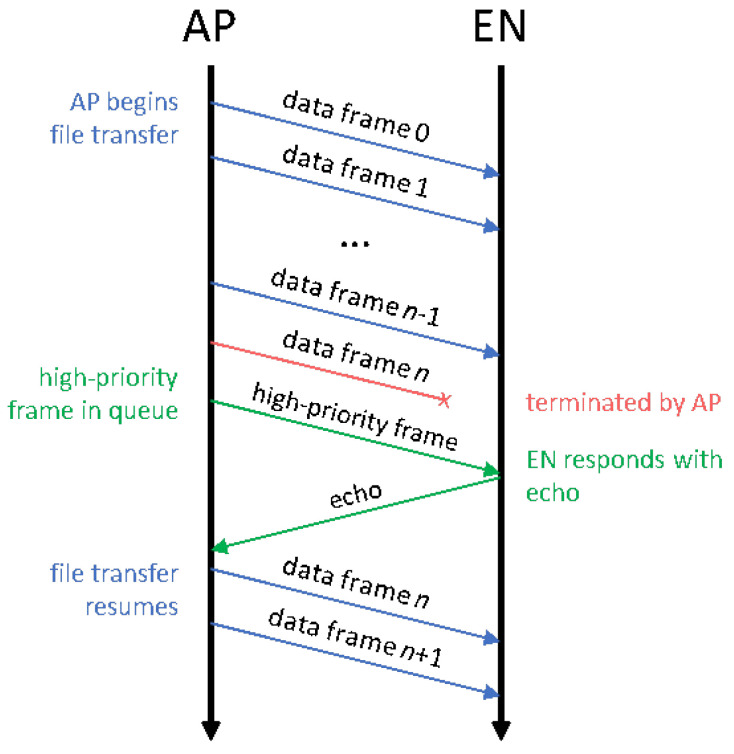
Message exchange process for timestamping.

Next, there is a category of messages that may or may not be considered critical based on the target application. The collection of health and status data from the AP to create a network aggregate may be considered critical if the ENs are operating in a harsh environment where part failure is common, allowing the AP to quickly react when an EN is not operating optimally. In more stable environments, this may not be considered critical. Lastly, we generally believe that the transfer of bulk data (e.g., files) should be treated as a low-priority process. A transfer of a 100 KB file using the ‘Data (medium)’ message type would take around 11 s to complete, but we want high-priority traffic to have the opportunity to flow freely and not wait the full 11 s for the file transfer to complete. A capability to terminate any outgoing low-priority frame is necessary to ensure the timely delivery of critical high-priority messages. Given that $\tau_{Sched}=0$, the high-priority message is immediately sent out over the wireless link; this is a major benefit of the RA-CDMA network construction. While the strong message correction ability of polar codes may allow a terminated message to be recovered at the receiver, more than likely, the sender will simply re-attempt transmission as soon as the high-priority frames have been delivered. Figure 4 shows a sample protocol diagram for the discussed scenario.

**Figure 5:**
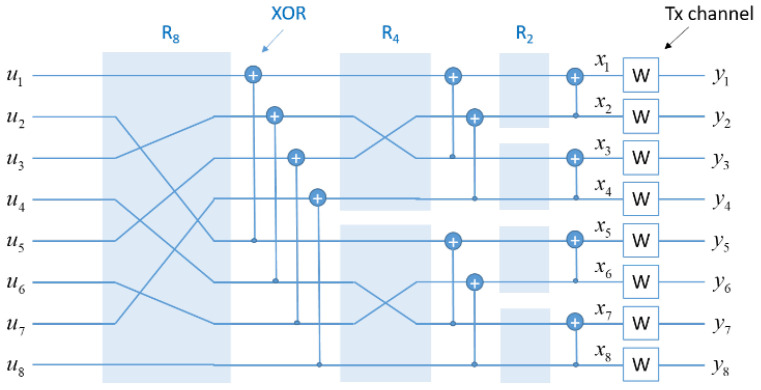
Polar encoder structure.

The anticipated reliability of the RA-CDMA-based HOPS design must also be considered. Fixed chip rate and spread ratio parameters yield specific throughput figures (Table 2) but also project an anticipated receiver operating point at −8 to −10 dB signal-to-noise ratio (SNR). Despite this, the FEC coding gain enhances performance with a relatively low computational cost. The encoding process involves straightforward two-input two-output XOR nodes, organized in a recursive structure resembling a fast Fourier transform (FFT) signal flow graph (as shown in Figure 5). This recursive polar encoder, with $N\log_{2}N$ operations, efficiently constructs codewords, taking 10 stages for $N=\begin{array}{c} 2^{10} \end{array}=1024$ instead of a resource-intensive 1024-by-1024 generator matrix operation.

The coding performance of a polar encoder results from the concept of frozen bits, known during coding and decoding, often set as zeros. Their importance lies in decoding, where their a priori knowledge imposes constraints on the process. The encoder takes a block of *N* data bits, comprising *k* information bits and N−k frozen bits, resulting in code rate R=k/N. Designing a polar code of rate *R* involves selecting frozen bit indexes. While various optimization approaches exist, a straightforward method results in the best performance [43]. This design relies on determining capacity bounds for bit channels in the polar structure, assuming a binary erasure channel (BEC) with erasure probability $P_{e}=0.5$. This worst-case scenario is also applied to the binary additive white Gaussian noise (AWGN) channel, with $P_{e}=0.5$ equivalent to $RE_{b}/N_{0}=-1.59$ dB, termed the design SNR. Despite better performance at 0 dB design SNR [43], we maintain $P_{e}=0.5$ for consistency.

**Figure 6:**
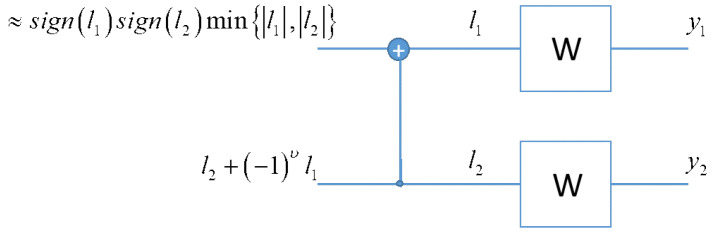
Log-likelihood ratio evaluations in the SCD.

Decoding polar codes involves traversing an inverted structure compared to the encoder (see Figure 6). The current approach favors the computationally efficient successive cancellation decoder (SCD) [42,44]. The decoding process begins with observations $y_{i}$ and, for a binary AWGN channel, the log-likelihood ratio (LLR) is calculated as $l_{i}=-2y_{i}\sqrt{2RE_{b}/N_{0}}$. LLRs are employed to limit the dynamic range and symmetrically center the decision statistic around zero. The LLR at XOR nodes is approximated as $l_{i}\approx \mathrm{sign}\left(l_{1}\right)\mathrm{sign}\left(l_{2}\right)\min(|l_{1}\left|,\right|l_{2}\left|\right)$, a computationally efficient and accurate term. At the decision point, if the LLR is positive, the decoded bit is zero, and vice versa. SCD decodes bits successively from lowest to highest index. Notably, the LLR expression at channel inputs is linear in observed output values, and a multiplicative factor uniformly affects LLR values throughout the structure. This property is advantageous for hardware implementation, allowing for the scaling of LLRs in a stage without impacting bit decisions.

**Figure 7:**
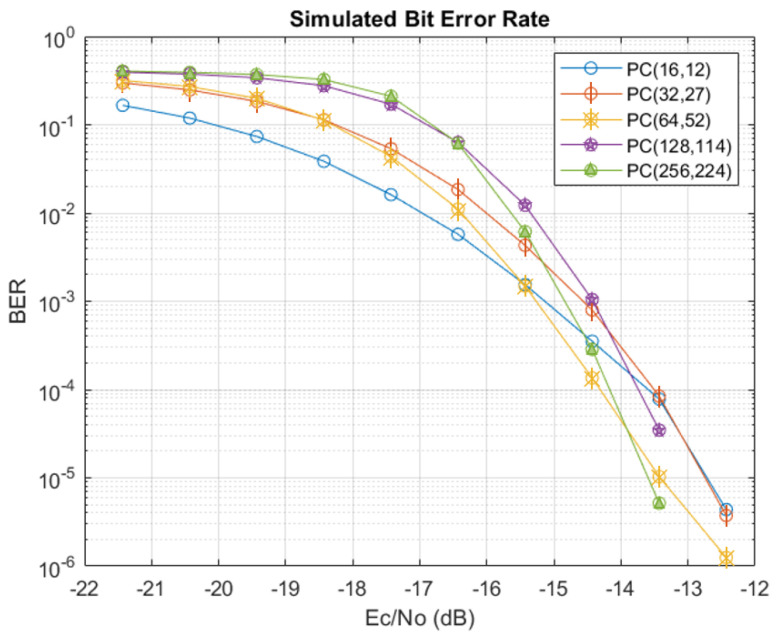
Simulated BER performance for each of the available message types.

**Figure 8:**
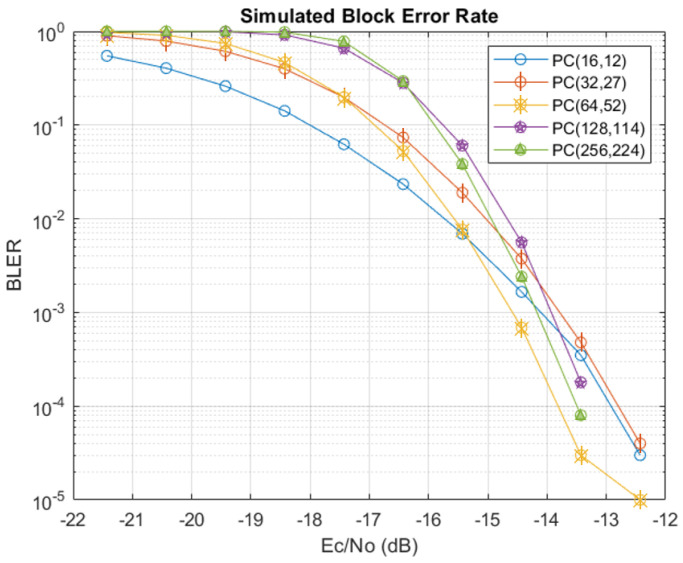
Simulated BLER performance for each of the available message types.

To give an idea of the expected performance for the polar-code-based SCD, simulations were performed in MATLAB R2020b using codeword blocks constructed according to the message types defined in Table 2. The frozen bit indices were determined as discussed before and correspond to the {4,5,12,14,32} indices with the lowest capacities. Figure 7 and Figure 8 show the performance for the HOPS simulated channel in terms of the bit error rate (BER) and block error rate (BLER), respectively, taken according to the energy per chip relative to the noise power spectral density (PSD). Simulations for each message type were run until $1\times 10^{6}$ blocks were evaluated for error. Note that there were no errors per 1 × 10^6^ blocks for $E_{c}/N_{0}$ values above −12 dB, so the x-axis is stopped there. Also note from Figure 8 that the smallest block size (code rate R=12/16) passes the reliability metric of $1-10^{-5}$ (alternatively, $\mathrm{BLER}=10^{-5}$) at a lower SNR when compared to the other message types, reflecting that fewer of the poorer capacity bit channels are used. However, it is very encouraging to see that all message types are expected to be reliable for $E_{c}/N_{0}>-12$ dB.

## 4. Quantitative Performance Testing

To evaluate our RA-CDMA HOPS prototype design with TSN extension capabilities, we engineered a test utility that exercises all components involved in the scheme and collects performance metrics. The utility initiates a generic data transfer from the AP to the EN using the low-priority transmit buffer. As the transfer is in progress, the AP periodically writes high-priority messages to the high-priority transmit buffer. The AP continues the data transfer while monitoring its receive buffer for an echo of the high-priority message. Once the echo is received, the AP compares the timestamp read from the received packet to one recorded at the time of original transmission. This gives us a round-trip time of high-priority packets amidst background traffic on the HOPS link. By utilizing the different messages available in the MAC, we emulate varying levels of channel congestion via background traffic and measure the high-priority round-trip time in these scenarios. Ultimately, however, since the PHY is able to terminate the active transmission of a low-priority message in favor of a high-priority one, this background traffic has virtually no effect on the observed latency.

In our testing, we ran the utility several times, once for each of the ‘data’ message types (extra short–extra long). Each test run included a continuous transfer of low-priority frames, with high-priority frames inserted at random intervals until 10,000 echoes had been received by the AP. In the AP, transmit timestamps are read from an FPGA register and recorded in software immediately prior to writing a frame to the high-priority transmit buffer. Upon receipt of a high-priority echo, another timestamp is appended to the received frame’s metadata within the demodulator (PHY). This receive timestamp is recorded at the same index as the corresponding transmit timestamp once the frame is pulled in for processing by the MAC. Table 3 provides the statistical results for each message type in terms of the one-way latencies for the injected high-priority frames. A key observation here is that the RA-CDMA prototype design can achieve sub-millisecond one-way latencies with the extra-short and short message types, while all of the other message types achieve from 1 ms up to 3 ms. The jitter is measured as the difference in maximum and minimum latencies, and is of the order of 100 µs for all message types.

**Figure 9:**
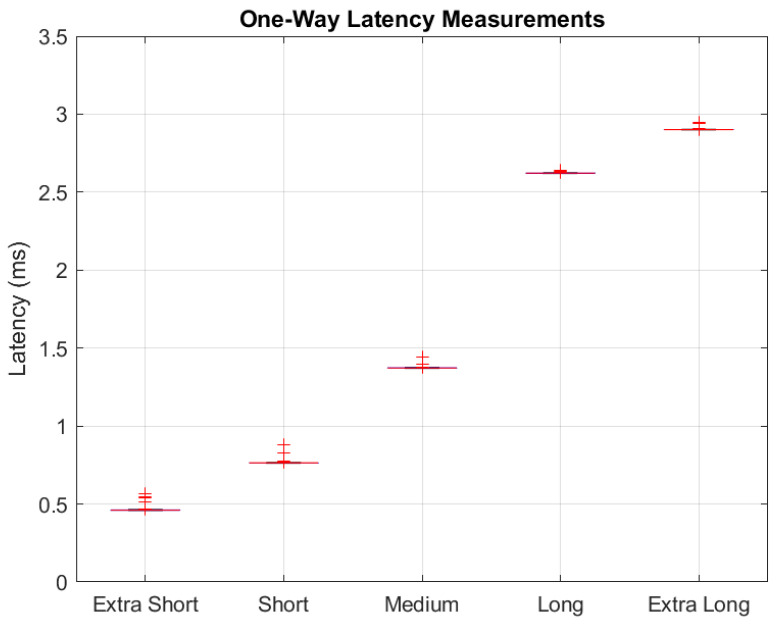
Boxplot of HOPS latency testing results.

**Figure 10:**
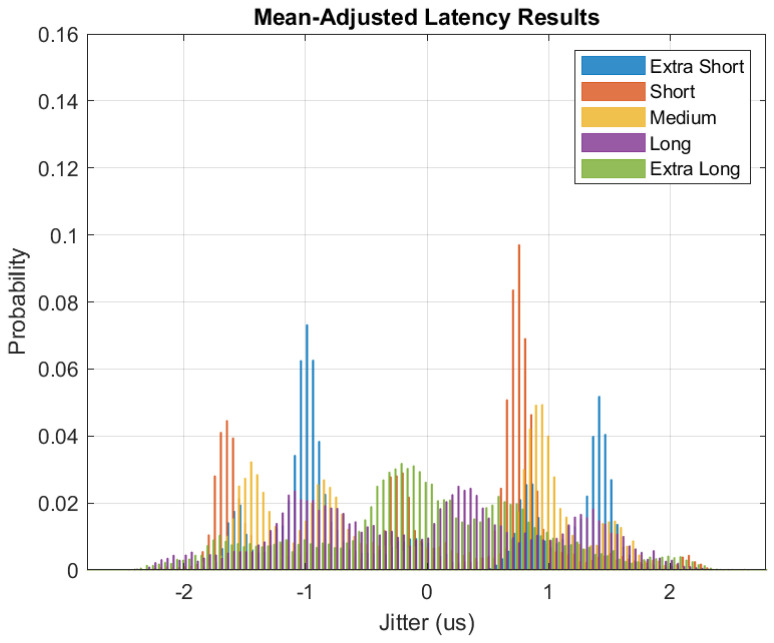
Histogram of HOPS latency testing results.

These same results are presented as a boxplot in Figure 9. Of particular note, the progression of increasing latency measurements aligns with what we expected prior to testing (larger message sizes correspond to larger latencies). By achieving one-way transmission of under 1 ms, the practical conclusion is that a reliable TSN extension may be achieved by doubling the prevailing 1 ms latency target of [1] rather than the prevailing methods that generally require order(s) of magnitude increase. Therefore, we contend that extending TSN expectations to include a wireless EN layer is feasible and warranted. Figure 10 offers a closer look at the measured jitter, visualized as a histogram of mean-adjusted one-way latency results. In our design, the jitter can be traced to a mixture of (1) data frame polling and insertion timelines, (2) receiver-side backlog of FEC decoding during high data flows, and (3) clock domain crossings, where the exact timing of when the MAC takes in a new received message depends on the state of the clocks when the message is made available in the buffer.

**Figure 11:**
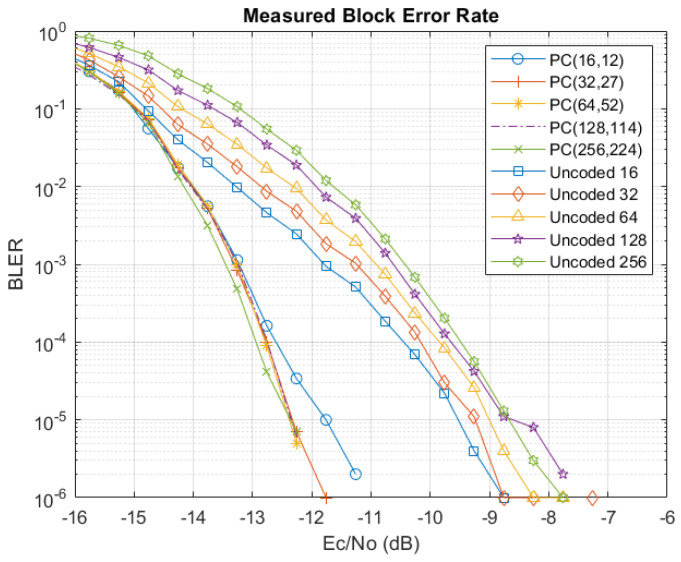
Measured BLER for each of the message types.

Figure 11 shows the measured BLER for each of the message types, along with the corresponding uncoded blocks, to demonstrate the improvements due to the polar-based FEC. At a BLER = $10^{-6}$, the FEC coding gain is roughly 3–4 dB for all message lengths, pushing the operating point of the signal down further into the noise from approximately −8 dB to −12 dB $E_{c}/N_{0}$. This is a significant improvement for a modest increase in computational resources compared to the uncoded baseline. Moreover, as shown in the latency and jitter measurements discussed previously, the polar-code-based decoder has a deterministic latency for all received message blocks, whether there are errors present or no errors, which is taken from the number of clock cycles required to complete the recursive SCD calculations. Other FEC block decoders, such those of Reed–Solomon (RS) codes, are not consistent in terms of latency, whereby errored blocks take significantly more clock cycles to perform the bit corrections. We can summarize that our RA-CDMA-based HOPS implementation offers significant flexibility to the IIoT application, specifically those that require a TSN-like deterministic wireless transmission of high-value data payloads with (sub-)ms latencies, minimal jitter, and high reliability, as supported by these BLER results.

## 5. Limitations

With the end goal of a wireless TSN implementation that is highly scalable and/or has lower power than existing technologies, the latency, jitter, and reliability measurements presented in this paper go a long way to establishing the feasibility of such a system. Full TSN operations, however, require accurate time synchronization throughout the system, as well as conformity to the requirements of 802.1Q. The work presented in this paper does not demonstrate a method for distributing the time across the network, though related work [45] has also demonstrated HOPS’ viability as a precision timing transfer waveform.

Bridging the wireless HOPS network to the traditional wired TSN network backhaul needs to be examined. In particular, given the constraints of spread spectrum signaling, there is a practical bound to throttling data rates up without giving up the interference mitigation benefits that enable RA-CDMA. As such, our models with a 10 MHz spread signal extend to a suggested bound for aggregate network traffic goodput/spectral efficiency of the order of 0.3 bits/Hz/s and an individual node goodput of around 0.01 bits/Hz/sec in networks of 100 nodes. These figures will vary up or down based on the duty cycle of transmissions.

## 6. Conclusions

This paper presents a wireless TSN extension geared toward IIoT applications based upon the secure and highly scalable RA-CDMA-based HOPS waveform. Our testing revealed excellent performance in the reliable and rapid delivery of high-priority traffic in the midst of varying levels of induced lower-priority user data traffic. Results measured from our hardware prototype, constructed on the IntelFPGA Arria 10 platform, show that (sub-)millisecond single-hop latencies can be achieved for each of the available message types, ranging from 12 bits up to 224 bits of payload. The latency of our implementation is on par with the lowest latency found in our literature search [32], which is also in the range of a few hundred microseconds. By achieving one-way transmission of under 1 ms, a reliable wireless TSN extension with comparable timelines to 802.1Q and/or 5G is achievable and proven in concept through our hardware prototype. Such a wireless TSN extension offers many new flexibilities in IIoT architectures, offering the potential to eliminate bulky cable harnesses even in applications that require the timing constraints traditionally only achievable by wired networks.

More research is needed to determine the feasibility of using the RA-CDMA-based HOPS waveform for time synchronization. The low-latency, low-jitter characteristics of HOPS certainly indicate that good results may be achieved. A time alignment method, such as that described in [31], should be developed and explored in future work. Based on the jitter results obtained in this study, absolute time differences of the order of microseconds could be expected. Moreover, given the arbitrary-phase nature of the waveform, the received phase angle could be incorporated along with the round-trip time (RTT) to derive a precise time synchronization method. Other work to consider pertains to the transmission of multiple high-priority frames simultaneously. The deeply spread nature of arbitrary-phase RA-CDMA signals allows two or more frames to be digitally combined by summing the individual samples prior to the data converter. The resulting transmission would consist of two overlapping high-priority frames that would be able to be sent without waiting for the outgoing high-priority frame to conclude.

## Figures and Tables

**Table 1:** Comparison of prior works attempting to bring TSN-like functionality to the IIoT.

Reference	Source	Technology	Summary	Year
[31]	MDPI Sensors	BLE	Novel time synchronization method achieved time differences of 69 ± 71 μs and 477 ± 490 μs on two hardware platforms. Ninety-fifth percentile of the errors was less than 1.8 ms.	2023
[32]	IEEE Transactions on Industrial Informatics	w-SHARP	Constructed a wired/wireless hybrid TSN network using w-SHARP and demonstrated a wireless latency of the order of 100 μs.	2022
[18]	IEEE Transactions on Industrial Informatics	IEEE 802.11	Proposed a novel precise wireless network time synchronization method; std. dev. of error < 500 ns.	2021
[33]	IEEE IDAACS-SWS	DECT ULE	Implemented proof of concept of the underlying DECT ULE portable parts configuration protocol.	2020
[19]	MDPI Energies	5G/IEEE 802.11	Surveyed research trends in wireless TSN, namely 5G, 802.11ax, and 802.11be.	2021
[27]	MDPI Electronics	5G	Integrated 5G URLLC into a TSN system, achieving <8 μs time synchronization error and end-to-end latency of <8 ms.	2022
[20]	IEEE Internet of Things Magazine	IEEE 802.11	Proposed a modification of standard 802.11 techniques with the addition of an early termination feature. Simulations show TSN latencies below 1 ms.	2023
[21]	MDPI Sensors	IEEE 802.11be	Discussed key features of Wi-Fi 7 and how they may be used to implement TSN functionality.	2021
[28]	IEEE Communications Standards Magazine	5G	Explained how 5GS Release 16 may be integrated into existing industrial networks; observed that a 5G bridge can support latencies of 1 ms and below.	2022
[29]	MDPI Telecom	5G	Focused on 5GS Releases 17 and 18, and the integration with TSN. Introduced a modification of the synchronization technique to achieve accuracy within 0.01 μs to 1 μs.	2024
[22]	MDPI Sensors	IEEE 802.11ac	Concluded that many of the benefits of TSN can be achieved within wireless systems. Noted that unexpected delays occur due to the queueing/scheduling of TSN frames due to retransmissions, random backoff times, and beacons.	2023
[23]	MDPI Sensors	RT-WiFiQA	Proposed RT-QoS and FGA for TDMA-based 802.11 systems in the developed RT-WiFiQA protocol. The results show that deterministic bounded latency is possible and reliability is improved.	2022
[24]	IEEE Open Journal of the Industrial Electronics Society	HAR^2^D-Fi	Modified standard 802.11 for reliable and deterministic communication; incorporated hybrid channel access mechanisms and temporal redundancy techniques. Changes were only made in the MAC layer.	2020

**Table 2:** Message type descriptions and FEC parameters.

Message Type	Symbols (w/ Preamble)	Block Size (N)	Information Bits (K)	Frozen Bits (N-K)	SCD Latency (Cycles)	Frame Duration (ms)	Max Throughput (kbps)
Extra Short	16	16	12	4	287	0.28	42.857
Short	24	32	27	5	741	0.42	64.285
Medium	40	64	52	12	1715	0.70	74.285
Long	72	128	114	14	4296	1.26	90.476
Extra Long	136	256	224	32	9885	2.38	94.117

**Table 3:** Summary of high-priority traffic one-way latency results.

Message Type	Mean	Std. Dev.	Variance	Max.	Min.	Jitter
Extra Short	461 μs	2.03 μs	4.13 μs	568 μs	458 μs	110 μs
Short	764 μs	1.72 μs	2.97 μs	879 μs	761 μs	118 μs
Medium	1.37 ms	1.35 μs	1.81 μs	1.44 ms	1.37 ms	73 μs
Long	2.62 ms	1.07 μs	1.14 μs	2.64 ms	2.62 ms	21 μs
Extra Long	2.90 ms	1.20 μs	1.43 μs	2.94 ms	2.90 ms	48 μs

## Data Availability

The raw data supporting the conclusions of this article will be made available by the authors on request.

## Abbreviations

The following abbreviations are used in this manuscript:

AP	Access Point
AVB	Audio/Video Bridging
AWGN	Additive White Gaussian Noise
BPSK	Binary Phase Shift Keying
C&C	Command and Control
BEC	Binary Erasure Channel
BER	Bit Error Rate
BLE	Bluetooth Low Energy
BLER	Block Error Rate
BW	Bandwidth
CDMA	Code Division Multiple Access
CSMA	Carrier-Sense Multiple Access
CSMA/CA	CSMA with Collision Avoidance
EMCON	Emissions Control
EN	Edge Node
FDMA	Frequency Division Multiple Access
FEC	Forward Error Correction
FFT	Fast Fourier Transform
FPGA	Field-Programmable Gate Array
GCL	Gate Control List
GTS	Guaranteed Timeslot
HOPS	High-Order PSK Signaling
IIOT	Industrial Internet of Things
LLR	Log-Likelihood Ratio
MAC	Medium Access Control Layer
PHY	Physical Layer
PSD	Power Spectral Density
PSK	Phase Shift Keying
PTP	Precision Timing Protocol
QPSK	Quadrature Phase Shift Keying
RA-CDMA	Receiver-Assigned CDMA
RTT	Round-Trip Time
SCD	Successive Cancellation Decoder
SNR	Signal-to-Noise Ratio
SPMA	Statistical Priority-Based Multiple Access
TDMA	Time Division Multiple Access
TRANSEC	Transmission Security
TSN	Time-Sensitive Networking
URLLC	Ultra-Reliable Low-Latency Communications
WSN	Wireless Sensor Network
